# Evaluating frailty in Medicaid Home and Community-based Services clients: a feasibility and comparison study between the SHARE-FI and SPPB

**DOI:** 10.1186/s40814-019-0429-2

**Published:** 2019-03-20

**Authors:** Margaret K. Danilovich, Laura Diaz, Colton Johnson, Erin Holt, Jody D. Ciolino

**Affiliations:** 1 Department of Physical Therapy and Human Movement Sciences, Northwestern University, 645 N. Michigan Suite 1100, Chicago, IL 60611 USA; 2 Department of Preventive Medicine, Northwestern University, 680 N. Lake Shore Drive Suite 1400, Chicago, IL 60611 USA

**Keywords:** Frailty, SHARE-FI, SPPB, Medicaid HCBS, Fried

## Abstract

**Background:**

Frailty assessment most commonly occurs within health care settings by health care providers. Implementing frailty assessment within non-medical settings that provide comprehensive social services for older adults may be an opportunity for population-based frailty screening and care. One such non-medical setting in which older adults receive care is Medicaid Home and Community-based Services (HCBS). Determining the feasibility of frailty screening within this non-medical setting is the first step towards population-based frailty assessment and care. The aims of this study were to (1) determine the feasibility of evaluating frailty using two different approaches (the Survey of Health Among Retired Europeans-Frailty Instrument (SHARE-FI) and Short Physical Performance Battery (SPPB)) among HCBS clients, (2) determine the agreement between the methods, and (3) explore specific frailty deficits on these measures to provide detailed knowledge on HCBS client impairments.

**Methods:**

This cross-sectional study occurred in HCBS client homes throughout the Chicagoland area. A research assistant with no health care provider training conducted all frailty assessments. We used the freely available SHARE-FI calculator to generate both a categorical and continuous frailty score. We used the SPPB to capture both a categorical score with frailty categories assigned as 0–6 (frail), 7–9 (pre-frail), and 10–12 (non-frail) and continuous score. We evaluated feasibility via domains of acceptability, implementation, adaptation, and practicality. We used Cohen’s kappa and Spearman’s correlation to determine agreement between frailty tools.

**Results:**

We enrolled *n* = 139 HCBS clients. Frailty assessment was feasibility via both the SHARE-FI and SPPB. The SHARE-FI was more practical given the fewer training needs, shorter administration time, and reduced equipment needs. There was a statically significant fair agreement between SHARE-FI and SPPB categorical scores with stronger agreement between SHARE-FI and SPPB continuous scores (*r* = − 0.448, *p* < .005; 95% CI, − 0.571, − 0.305). Among the five frailty criteria on the SHARE-FI, a pattern emerged of the highest frequency of positive responses to each criterion among frail clients followed by pre-frail and then non-frail.

**Conclusions:**

Frailty assessment is feasible within HCBS settings conducted by a non-medical provider. Using continuous frailty scores provides stronger agreement between measures compared with categorical scores. Frailty can be feasibly measured in a non-medical setting providing initial evidence for a mechanism for population screening and care for frailty in HCBS.

## Background

One of the ways in which older adults with physical or cognitive impairments age-in-place is through the use of Medicaid Home and Community-based Services (HCBS) which provide in-home care services (e.g., home care aides, emergency response services, adult day programs, meals) in lieu of nursing home placement. Clients are often anecdotally described as “frail” [1], yet frailty assessment is not currently a component of routine client evaluations despite the recommendation that all adults over the age of 70 be evaluated yearly for frailty [2]. Further, as frailty assessment is often implemented by health care providers and conducted individually, implementing frailty assessment through HCBS provides an opportunity for population-based screening.

Despite the prevalence [3], adverse health outcomes associated with [4], and recommendation for screening [2], there is currently no gold standard for frailty assessment. Over 60 frailty measures exist ranging from fully subjective patient self-report measures to hybrid subjective and objective assessments to health care provider observation [5]. Frailty measures vary in equipment requirements, and time to administration ranges from 5 to 30 min. Given the wide variability in measure scope, there is little guidance on the optimal measure for frailty assessment among older adult HCBS clients.

Originally designed for primary care settings with an administration time less than 5 min, the Survey of Health Among Retired Europeans-Frailty Instrument (SHARE-FI) evaluates physical frailty based on constructs from Fried’s frailty phenotype criteria [6]: fatigue, appetite changes, weakness, walking difficulties, and frequency of physical activity participation. Based on the respondent’s self-report of deficits in these constructs and a grip strength measurement, a composite frailty score is generated and that score classifies individuals as non-frail, pre-frail, or frail. In contrast to the phenotype model of frailty, the Short Physical Performance Battery (SPPB) objectively assesses physical function relative to three sub-scales: gait speed, static balance, and lower body strength. Each subscale is scored from 0 to 4 with a total possible score of 12. While not originally designed as a frailty assessment measure, total scores of less than 9 have been used as a cut point for frailty classification [7].

Frailty assessment has the potential to assist in four areas of patient care: (1) understanding the biology of the condition, (2) diagnosis and care planning, (3) evaluating response to intervention, and (4) risk stratification [8]. Given that the most recommended treatment for frailty is not medication, but rather physical activity, nutrition, care coordination, and disease self-management, the HCBS setting provides an opportunity for frailty assessment given that HCBS provide care coordination, nutrition services, and physical activity. Frailty assessment in HCBS could assist with care planning by providing a mechanism to allocate resources to those most frail and in need of assistance, as an outcome measure to evaluate client response to HCBS interventions, and to identify clients at greatest risk for adverse health outcomes and subsequent greater health care expenditures. However, before widespread uptake of any frailty assessment in HCBS settings can be recommended, the feasibility of frailty assessment in this setting must first be determined. Given the lack of information regarding frailty assessment within HCBS, the primary aim of this study was to determine the feasibility of evaluating frailty using two different approaches (SHARE-FI and SPPB) among HCBS clients. We selected the SHARE-FI given its short administration time and use of only one physical performance measure, as we thought these factors would make for easy administration by lay personnel in HCBS. We chose the SPPB because it is one of the most widely used objective measures of functioning in older adults and recent papers have reported the use of the SPPB as a frailty screening tool [7]. Given the non-medical care provided in HCBS, we did not choose frailty tools such as the Frailty Index that inquire about medical or clinical deficits or the Clinical Frailty Scale that requires clinicians to make a judgment or clinical decision about a person’s functioning. Our secondary aim was to determine the prevalence of frailty among HCBS clients using these tools and determine the agreement between the methods. Finally, we aimed to explore specific deficits on these measures to provide new knowledge on the impairments of HCBS clients.

## Methods

### Design and setting

We used a cross-sectional study design involving 139 HCBS clients from Help at Home, Inc.’s Chicago, Illinois office enrolled in an ongoing exercise trial [9]. All clients signed informed consent, and data collection occurred in client’s homes following the same scripted instructions and equipment (e.g., standard-height chair, hand-held dynamometer, rope that marked distance for gait speed testing) that one research staff member brought to each home to ensure testing procedure standardization. The research staff member collecting data was not a health care provider and had no prior experience collecting any physical assessments of older adults.

### Participants

In the state of Illinois, Medicaid HCBS are offered through the Community Care Program. Persons are eligible for the Community Care Program if they are at least 60 years of age, have net assets less than $17,500 USD, and require nursing home services as determined by a case manager. We recruited English-speaking participants receiving home care aide assistance with a Telephone Interview for Cognitive Status [10] score above 25. Each client received $10 for study participation.

### Measurements

#### Feasibility of frailty assessment

To evaluate the feasibility of frailty assessment using the SHARE-FI and SPPB, we assessed the acceptability, implementation, adaptation, and practicality [11]. We defined acceptability as the number of clients who could participate in frailty assessment, implementation as the extent to which clients could complete frailty assessment without modifications, adaptation as modifications that occurred to evaluate frailty in the client’s home, and practicality as the time and resources involved in frailty assessment.

#### SHARE-FI

We used the free online calculator specific for men and women to calculate both continuous and categorical frailty scores based on the answers to four self-report questions and grip strength measurement [12]. Fatigue was measured as a yes/no response to whether the respondent has too little energy to complete desired tasks. Appetite was measusured as self-reported food intake over the last month (diminished, same, or increased). Weakness was assessed by two repetitions of grip dynamometry on each hand using a Jamar dynamometer (Lafayette Instruments, USA). Walking difficulties were assessed by self-report of the ability to walk 100 m and climb one flight of stairs. Physical activity was measured via self-report of frequency of engagement in low to moderately intense physical activities.

#### SPPB

We used the freely available SPPB data collection form and provided a score between 0 and 4 for each sub-scale: gait speed, static balance, and chair rise. A final summary score out of 12 was calculated, and we categorized clients using the following cutoffs: 0–6 (frail), 7–9 (pre-frail), and 10–12 (non-frail) [13].

#### Additional data collection

We collected participant demographic information including age, race, sex, level of education, housing environment, living arrangement, and comorbidities.

### Data analysis

We summarized client demographics and assessment scores using mean ± standard deviation (or median and inner quartile range [IQR] where appropriate) for continuous variables and frequency/percent for categorical variables. We used Cohen’s kappa statistics with relevant confidence intervals to evaluate agreement between SHARE-FI questions and the Cohen’s kappa statistics and Spearman’s correlation coefficients to evaluate agreement between SHARE-FI questions and SPPB sub-scales. We used independent two-sample *t* tests to evaluate differences in gait speed among those reporting difficulty walking on the SHARE-FI. To evaluate relationships between categorical variables (e.g., frailty category and fatigue), we used chi-squared tests (or Fisher’s exact tests in the cases of small cell counts). All statistical tests assumed a two-sided, 5% level of significance, and we used SPSS Version 25 for analysis.

## Results

### Frailty assessment feasibility

All 139 clients completed SHARE-FI testing, and none refused to complete any items or answer any self-report questions demonstrating high acceptability of this frailty assessment. Five clients had hand issues from a previous stroke or arthritis that made them unable to complete the grip strength measurement on one side. Those clients received a grip strength score of 0 kg because they were unable to demonstrate any grip strength following the standardized testing instructions. We adapted the SHARE-FI question on walking for US clients by providing the distance in feet in addition to meters. Conducting the SHARE-FI was practical as it took less than 5 min per person, and the only equipment required was a hand grip dynamometer with an approximate cost of $200 USD. The only training required for the frailty assessor to administer the SHARE-FI was in grip strength measurement, which took less than 30 min to provide training.

Likewise, the SPPB showed high client acceptability and no clients refused testing. However, 17 clients (12.2%) were non-ambulatory and could not complete the gait speed test, 58 clients (41.7%) could not perform the chair rise test following standardized instructions to rise without using their arms, and 17 clients (12.2%) received 0 points on the balance test for not being able to maintain static standing for 10 s. To implement the SPPB using standardized equipment, the research assistant transported the same standard-height chair to each client’s home. No adaptations to the SPPB were necessary for in-home testing, although the research assistant occasionally needed to move furniture to clear a path in the home for the gait speed test. Of the 58 clients unable to complete the chair rise test following standardized instructions, 22 clients were able to perform the chair rise if allowed to use their arms; however, we did not score this modification or use it in analysis. Time to administer the SPPB varied between 5 and 10 min per client depending on the client’s need for rest in between sub-scale assessments. While able to be administered in-home, the SPPB required a standard-height chair brought to each visit, stopwatch, and a cleared 3 m walking space for testing. The training required for the frailty assessor to administer the SPPB took approximately 2 h and included instruction in guarding techniques during balance and gait to prevent falls. There were no adverse events or losses of balance during frailty assessment on either the SHARE-FI or SPPB.

### Frailty demographics and agreement between SHARE-FI and SPPB

Table 1 displays client demographics. The most common comorbid conditions were visual problems (78%), hypertension (77%), and arthritis (72%). Overall, 45% of clients were frail, 35% pre-frail, and 20% non-frail based on SHARE-FI scores. Using SPPB scoring criteria of 0 to 6 (frail), 7 to 9 (pre-frail), and 10 to 12 (non-frail) [13], 69% of clients were frail, 28% pre-frail, and 3% non-frail based on SPPB scores. We used Cohen’s kappa to investigate the agreement between SHARE-FI and SPPB frailty categories (e.g., frail, pre-frail, and non-frail). There was fair, but statistically significant agreement between these measures (*κ* = 0.264 *p* < 0.001). When using the continuous scoring version of the SHARE-FI and SPPB, there was a statistically significant and moderate agreement in scoring (*r* = − 0.448; 95% CI, − 0.571, − 0.305; *p* = 0.006).

**Table 1:** Client demographics

	Mean (std dev) or *N* (%)
Age	74.19 (8.36)
Gender	
Female	109 (78.42)
Male	30 (21.58)
Race	
Other	9 (6.47)
African American	130 (93.53)
Housing environment	
House	42 (30.22)
Apt/townhome/condo	48 (34.53)
Senior living building	49 (35.25)
*N* comorbidities^a^	5 (4–6)

^a^Median (IQR)

We further investigated the agreement between analogous constructs (i.e., subcomponents) between SHARE-FI and SPPB. We defined overlapping frailty constructs as shown in Table 2. We first looked at the association between SHARE-FI walking difficulties and SPPB gait test scores using an independent two-sample *t* test. The average gait speed for participants who reported no walking difficulty was significantly faster by 0.22 m/s than those with self-reported walking difficulties [95% CI, 0.12, 0.32; *p* < 0.001]. To compare SHARE-FI weakness and SPPB chair rise (a proxy measurement for strength) scores, we first created a weakness variable for each hand by averaging the two trials of grip strength. We used the hand with the larger average for analysis. We then used Spearman’s correlation test to determine the relationship between SHARE-FI weakness and SPPB chair rise. There was a weak, positive association between SHARE-FI grip strength and SPPB chair rise (*ρ* = 0.207; 95% CI, 0.042, 0.361; *p* < 0.05).

**Table 2:** Comparison of frailty constructs between Fried, SHARE-FI, and SPPB

Fried frailty phenotype	SHARE-FI	SPPB
Slow walking speed	Walking difficulties	Gait speed test
Weakness (grip strength)	Weakness (grip strength)	Chair stand test
Exhaustion	Exhaustion	n/a
Unintentional weight loss	Loss of appetite	n/a
Low physical activity	Low physical activity	n/a

### Frailty impairments on the SHARE-FI

Table 3 shows the percentage of clients reporting positive responses to SHARE-FI frailty criteria by frailty category. There was a significant difference in all five SHARE-FI frailty criteria between frail, pre-frail, and non-frail clients (*p* < .0001). A predictable pattern emerged whereby the highest frequency of the presence of each of the five frailty criteria (e.g., food intake, low physical activity, weakness, walking difficulties, or fatigue) was found among frail clients followed by pre-frail and then non-frail. Engagement in activities on the SHARE-FI had the most variability in responses. Of clients reporting frequent engagement in activities requiring low to moderate levels of energy (more than once a week), 34% were non-frail, 49% were pre-frail, and 16% were frail. We then explored the relationship between this construct and other criteria. The chi-square test did not provide evidence of a significant relationship (*p* = .258) between exhaustion and engagement in activities. Among clients reporting exhaustion, 31% hardly ever or never engaged in activities that required low to moderate levels of energy, while 44% reported engaging in these activities more than once a week.

**Table 3:** Percentage of clients reporting positive responses to SHARE-FI frailty criteria by frailty category

	Overall (%) (*n* = 139)	Non-frail (%) (*n* = 28)	Pre-frail (%) (*n* = 48)	Frail (%) (*n* = 63)
Hardly ever or never engage in activities	23.02	3.45	10.34	86.21
Loss of appetite	28.57	5.56	22.22	72.22
Exhaustion	50	12.7	28.57	58.73
Difficulty climbing 1 flight of stairs	65.87	8.43	34.94	56.63
Difficulty walking 100 m	76.19	6.25	40.63	53.13
Grip (< 20 kg for females and < 30 kg for males)	52.52	25.00	39.58	77.78

## Discussion

This paper presents an evaluation of the feasibility of frailty assessment using the SHARE-FI and SPPB among Medicaid HCBS clients. Our findings show that SHARE-FI and SPPB assessment is feasible among HCBS clients and HCBS clients have high levels of frailty and physical function impairments regardless of type of frailty assessment employed. Unsurprisingly, when the SHARE-FI is measured on a continuous scale, its association with the SPPB is stronger since this method provides more information than the discrete categorization.

While both methods were feasible for assessing frailty, the SHARE-FI is a quicker and easier tool to administer, requires less equipment, and does not need as much space compared to the SPPB. However, the physical assessment provided by the SPPB did identify more clients as frail compared to the SHARE-FI evidenced in the greater prevalence of frailty among HCBS clients when using the SPPB (69%) compared to the SHARE-FI (44%). This suggests the SPPB may have greater sensitivity, but without a gold standard for frailty measurement, it is difficult to determine if the SPPB is overly sensitive or if SHARE-FI is not sensitive enough. To our knowledge, only one other study to our knowledge has investigated the relationship between the SPPB and another frailty assessment tool. Pritchard et al. found greater frailty prevalence when comparing the SPPB to the Fried phenotype [13]. Given that the SHARE-FI relies upon self-reported data for four of the five questions, it is not surprising that frailty may be under or overestimated compared with more objective assessment.

Weakness is evaluated by two vastly different methods in the SHARE-FI compared with the SPPB. The SHARE-FI evaluates hand grip strength to determine weakness while the SPPB evaluates the ability to rise from a chair as a proxy of lower extremity strength. Considering that loss of independence is a primary predictor of nursing home placement [14] and termination of HCBS services, evaluating weakness in frailty by a mechanism that more directly relates to physical functioning may be warranted. However, the SPPB does not capture elements of frailty such as unintentional weight loss and fatigue that are important components to capture in providing holistic care in the HCBS environment.

While the majority of clients live independently at home, they report high levels of exhaustion, difficulties walking, and stair-climbing deficits that may interfere with the ability to remain living independently. Further, there was a high prevalence of low levels of physical activity engagement, particularly among the frail. This is in concordance with other reported literature documenting the large amount of time in sedentary behavior among frail older adults [15, 16]. Interestingly, of participants who reported difficulty with stair climbing, many reported engaging in activities requiring a low to moderate level of energy more than once a week. It would be reasonable that assume those who participate in more frequent physical activity should actually have fewer mobility impairments and less difficulty with stair climbing. However, given the high prevalence of fatigue in the sample and the SPPB total scores indicating overall low levels of physical function, it may be that any activity, even basic activities of daily living, require low to moderate levels of energy, overinflating the self-reported physical activity score. As such, the SHARE-FI physical activity question may actually be more reflective of the respondent’s perception of fatigue than participation in leisure-time physical activity. Further research is needed to understanding the association between SHARE-FI physical activity self-reports and objectively measured physical activity data.

An important strength of this study was that all data were collected over a 2-week period, so we would not anticipate seasonality to influence self-report responses which, in the cold winter climate in which the study occurred, could dramatically influence physical activity. A limitation of this study was a small sample size based solely in an urban area and that we did not enroll significantly cognitively impaired clients because of the cognitive requirement needed to follow directions for frailty testing through both the SHARE-FI and SPPB. Thus, future research should explore the feasibility of measuring frailty through caregiver or family self-report for the population of Medicaid HCBS clients. Further, the SHARE-FI and SPPB only evaluate physical frailty. Particularly in the low-resource population of Medicaid HCBS, evaluating dimensions such as cognitive and social frailty is of importance.

## Conclusions

Our findings have important implications for practice, policy, and research in Medicaid HCBS. For service providers, our results demonstrate the feasibility of frailty assessment for HCBS clients and provide evidence that population-based frailty screening could occur in HCBS settings. Particularly for the SHARE-FI, frailty assessment can be accomplished easily through mostly self-report and a very simple objective physical measure. This quick tool to administer with minimal training requirements would provide a wealth of information to help guide care. As frailty assessment has been shown to provide critical additional information regarding risk for hospitalization, falls, and mortality, our findings suggest assessing frailty among HCBS clients may provide additional information with which to tailor interventions and direct care. As the current recommendation is that all older adults over the age of 70 be screened for frailty, our findings show that frailty assessment can be implemented and should be considered as part of the standard Medicaid HCBS screening for determining need for services.

## Abbreviations

HCBS: Home and Community-based Services
SHARE-FI: Survey of Health Among Retired Europeans-Frailty Instrument
SPPB: Short Physical Performance Battery
